# Porechop_ABI: discovering unknown adapters in Oxford Nanopore Technology sequencing reads for downstream trimming

**DOI:** 10.1093/bioadv/vbac085

**Published:** 2022-11-21

**Authors:** Quentin Bonenfant, Laurent Noé, Hélène Touzet

**Affiliations:** Univ. Lille, CNRS, Centrale Lille, UMR 9189 - CRIStAL—Centre de Recherche en Informatique Signal et Automatique de Lille, Lille F-59000, France; Univ. Lille, CNRS, Centrale Lille, UMR 9189 - CRIStAL—Centre de Recherche en Informatique Signal et Automatique de Lille, Lille F-59000, France; Univ. Lille, CNRS, Centrale Lille, UMR 9189 - CRIStAL—Centre de Recherche en Informatique Signal et Automatique de Lille, Lille F-59000, France

## Abstract

**Motivation:**

Oxford Nanopore Technologies (ONT) sequencing has become very popular over the past few years and offers a cost-effective solution for many genomic and transcriptomic projects. One distinctive feature of the technology is that the protocol includes the ligation of adapters to both ends of each fragment. Those adapters should then be removed before downstream analyses, either during the basecalling step or by explicit trimming. This basic task may be tricky when the definition of the adapter sequence is not well documented.

**Results:**

We have developed a new method to scan a set of ONT reads to see if it contains adapters, without any prior knowledge on the sequence of the potential adapters, and then trim out those adapters. The algorithm is based on approximate *k*-mers and is able to discover adapter sequences based on their frequency alone. The method was successfully tested on a variety of ONT datasets with different flowcells, sequencing kits and basecallers.

**Availability and implementation:**

The resulting software, named Porechop_ABI, is open-source and is available at https://github.com/bonsai-team/Porechop_ABI.

**Supplementary information:**

Supplementary data are available at *Bioinformatics advances* online.

## 1 Introduction

Oxford Nanopore Technology (ONT) is a versatile sequencing technology that produces long reads and has many applications, including *de novo* genome sequencing, metagenomics (Moss *et al.*, 2020), structural variants (Sakamoto *et al.*, 2021) and transcriptome sequencing (Sessegolo *et al.*, 2019; Workman *et al.*, 2019). One common feature of all sequencing kits and flowcells is that library preparation includes ligation of adapter sequences to both ends of DNA, cDNA or RNA fragments. These adapters facilitate strand capture and loading of a processive enzyme.

Since the adapters are sequenced with the fragment, this implies that resulting reads may contain full-length or partial adapters due to incomplete sequencing.

These extra sequences can be removed by *read trimming*. It allows one to deal with adapter contamination and to avoid unexpected interferences in downstream analyses. Indeed, read trimming leads to better contiguity in genome assemblies (Murigneux *et al.*, 2021), higher accuracy in RNA-seq reads clustering (De la Rubia *et al.*, 2022), to cite a few examples of application. Tools such as Porechop that finds and removes adapters, were designed to perform this task efficiently (Wick, 2017) and are widely used by the community. The main limitation however is that such tools rely on a static database of known adapters. This prerequisite can be a critical issue when the adapters used are not known, when they are not present in the database or when there is no information about the fact that the reads have already been trimmed out or not. In particular, Porechop database is no longer maintained since October 2018. More recently, ONT released the Guppy toolkit that contains several basecalling and post-processing algorithms, including adapter trimming. But, this toolkit can be seen as a black box with no control on the output. Moreover, it cannot be applied to previously published public datasets when the FAST5 files are no longer available.

In this context, it is therefore particularly useful to have tools that can deal with adapters of unknown origin. This problem has been recently addressed in Ranjan *et al.* (2022) that proposes an approach based on visual confirmation and input-assisted removal of adapter contamination.

In this article, we present an alternative way to deal with undocumented adapters. We have developed a new algorithm to automatically infer adapter sequences from raw reads alone, without any external knowledge or database. The method determines whether the reads contain adapters, and if so what the content of these adapters is. It uses techniques coming from string algorithms, with approximate *k*-mer, full-text compressed index and assembly graphs.

The method is available as an extension of the existing Porechop tool, and the resulting software is named Porechop_ABI (ABI stands for *ab initio*). This new tool is proving to be useful to clean untrimmed reads for which the adapter sequences are not documented and to check whether a dataset has been trimmed or not. It is even able to find leftover adapters in datasets that have been previously processed with Guppy with trimming mode activated or to deal with datasets with several distinct adapters.

## 2 Algorithm for adapter inference

The goal is to design a computational method that is able to infer, or to accurately guess, the adapter sequences from a set of untrimmed reads. The starting point of the method is that adapters are expected to be found mainly at each extremity on untrimmed reads and are over-represented sequences that could be distinguished from the biological content. To work properly, the method should fulfill several additional constraints: it should be tolerant of sequencing errors; it should scale to large datasets; it should deal with adapters of varying length (from 16 nt to more than 30 nt); it should accommodate to the presence of several distinct adapters in the dataset. For that, we have developed a new algorithm that is based on four main steps:

- Reads sampling: Select 10 independent samples of 40 000 reads from the dataset, then for each read of the samples select start and end regions of length 100 nt.
- Approximate *k*-mer counting: Find and count *k*-mers that are over-represented throughout the start (respectively end) region. This search allows for edit errors (insertions, deletions and mismatches).
- Adapter construction: Reconstruct the start (respectively end) adapter sequence by assembling *k*-mers using an assembly graph based on most represented *k*-mers.
- Consensus between samples: Align and compare the start (respectively end) adapters found for each of the 10 samples, and build a consensus sequence. When the sequences are not fully compatible, when there is no single consensus sequence, the method outputs several adapters associated with a support score that corresponds to the proportion of samples containing the adapter.

The algorithm is described in full details in Section S1 of Supplementary Material.

## 3 Implementation

The algorithm is implemented in C++ and Python, using the SEQAN library (Reinert *et al.*, 2017) and the NetworkX library (https://networkx.org/). SEQAN provides Optimal Search Schemes together with a bidirectional Burrows–Wheeler Transform and a FM-index for read indexing, which makes the search of approximate *k*-mers very efficient (Hauswedell, 2022; Kianfar *et al.*, 2018). NetworkX is a graph library that is used in the assembly step of the algorithm.

**Fig. 1.**
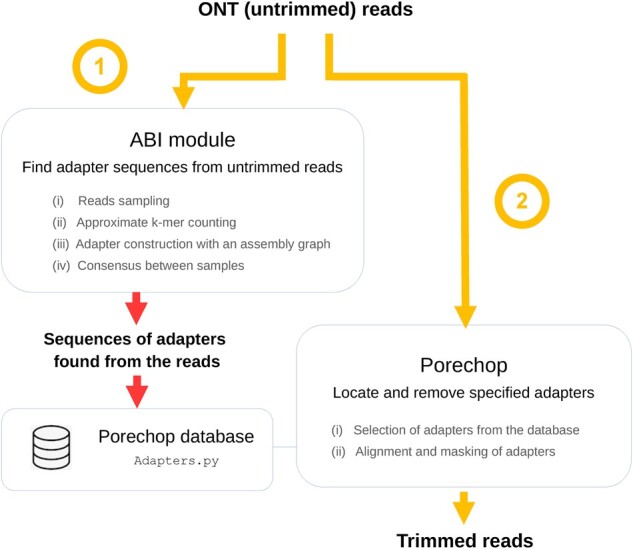
Organization of Porechop_ABI. (1) The tool takes as input a set of reads. When those reads are untrimmed, the ABI module is able to determine the sequences of the adapters that have been used in the sequencing protocol. (2) Those sequences are then ready to be used by Porechop for downstream trimming. If the reads are already trimmed out, no adapter sequence is returned by the ABI module

This new code is available as an extension of Porechop to form a new software: Porechop_ABI. The algorithm presented in Section 2 is implemented in the ABI module (*ab initio*), which is interfaced with Porechop. Porechop_ABI, as a whole, allows one to automatically infer adapters and trim them in a single run. In practice, adapter sequences found by the ABI module are loaded in the database used by Porechop (file adapter.py). This organization is summarized in Figure 1. It is also possible for the user to only run the ABI module. In this specific case, the output is simply a set a putative start adapters and end adapters, when such sequences are extracted from the raw reads.

Installation can be performed directly from the source code, or using the conda package management system from the bioconda channel.

## 4 Experimental results

We present the results of the software on a series of datasets, which are listed in Table 1. The complete source of each data along with its description is available in Section S2 of Supplementary Material, where we also provide additional details on experimental results and one more dataset from the Nanopore WGS Consortium composed of a human poly(A) transcriptome from B-lymphocyte cell line. In all experiments, Porechop_ABI was used with default parameters.

**Table 1.** List of tested datasets: the first dataset is composed of simulated reads (with the read simulator BadReads)

Organism, tissue	Type	Flowcell	Sequencing Kit	Base caller	Source
Mouse	cDNA	Simulated with BadReads			
*Eucalyptus pauciflora*	DNA	r9.5	SQK-LSK108	Albacore 2.0.2	SRR7153074.1
*Prunus dulcis*	DNA	r9.4	SQK-NSK007	Metrichor	SRA ERR3430401
Mouse brain	cDNA	r9.4	SQK-LSK008	Metrichor	SRA PRJEB25574
*Zea mays*	cDNA	r9.4	SQK-PCS108	Guppy	SRA PRJNA643165
*Percina kusha*	mtDNA	FLO-FLG001	SQK-LSK110	Guppy	SRA PRJNA742674

*Note*: The three following datasets are composed of cDNA and DNA reads for various organisms, flowcells, basecallers and adapter sequences. The two last datasets contain reads that have previously been processed with the basecaller Guppy and that are not supposed to exhibit adapter sequences.

**Fig. 2.**
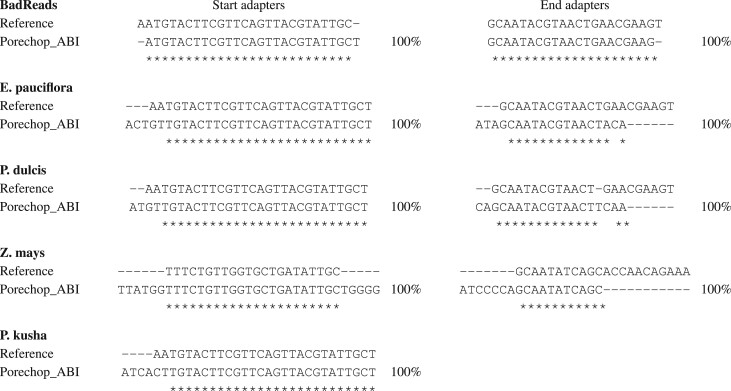
Start and end adapters for the BadRead, *E.pauciflora*, *P.dulcis*, *Z.mays* and *P.kusha* datasets. For each example, we searched for the sequences of the start and end adapters. The first sequence (top) is the reference adapter sequence, such as documented in the associated publication or found in the Porechop database. The other sequence (bottom) is the sequence determined *ab initio* by Porechop_ABI from the raw reads, without knowing the reference sequence. The percentage indicated with the Porechop_ABI sequence is the support score, computed during the sampling phase of the algorithm. Identical positions between the reference sequence and Porechop_ABI adapter are marked by stars

The first dataset is composed of artificial long reads generated using BadRead (Wick, 2019) from the reference assembly of the mouse transcriptome for the GRCm38 genome. Porechop_ABI finds one single sequence for the start adapter and one single sequence for the end region, both with support 100%. See Figure 2. Those two sequences each match with BadReads adapters: 26 out of 28 nt for the start adapter and 21 out of 22 nt for the end adapter. Regarding the end adapter, this result is particularly convincing because, according to BadReads specification, only half of the reads are intended to contain the end adapter, with a mean length of 20% of the original adapter across the whole dataset. Even in this case, Porechop_ABI is able to accurately recover the signal.

The second dataset is a high-coverage sequencing of the genome of a *Eucalyptus pauciflora* individual. Once again, Porechop_ABI finds a single start adapter sequence and a single end adapter sequence, both with support 100% (Fig. 2). As for the start region, the adapter found by Porechop_ABI is very similar to the top adapter of SQK-NSK007-Y: SQK-NSK007-Y-top is 28 nt long, and we correctly recovered 26 nucleotides at the 3′ end. Our sequence does, however, contain four extra-nucleotides at the 5′ extremity. To see if this difference has an impact on the subsequent trimming step, we compared trimmed reads with each one of the two adapter sequences. This comparison shows that only 5% of the total amount of reads have distinct trimming sites. Regarding the end region, the majority of reads (74%) have not been trimmed out with either the SQK-NSK007_Y_bottom adapter or the adapter found with Porechop_ABI. For the remaining reads, there is a strong overlap (more than 60%) between the two methods. We also used this dataset to estimate the stability of results across samplings. We ran Porechop_ABI 100 times independently on the whole dataset (818 267 reads). For the start adapter, all runs produced exactly the same output, with 100% support at each try. For the end adapter, there are some minor variations: 91 tests obtained the same sequence with maximal support (100%), 8 tests obtained the same sequence with a lower support (98.3%) and one test produced a sequence with one extra nucleotide (100% support). This shows that the sampling strategy is stable, and that the software can be trustfully used without re-sampling.

The third dataset is composed of genomic reads for the *Prunus dulcis*, the almond tree. Once again, Porechop_ABI finds one start adapter and one end adapter that both closely match the expected sequences and that are suitable for trimming.

**Fig. 3.**
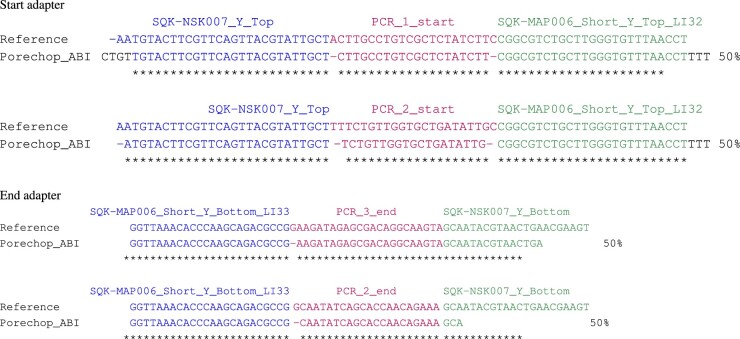
Start and end adapters for the mouse brain dataset

All those three first datasets were intended to contain only one start adapter and one end adapter. The fourth dataset, the mouse brain, was sequenced with a custom protocol for cDNA, and is supposed to contain multiple distinct adapters. In this context, Porechop_ABI identified two distinct sequences for the start region, both with support 50% (see Fig. 3). Those sequences share the same initial motif, which appears to be SQK-NSK007_Y_Top, and the same final motif, which is SQK-MAP006_Short_Y_Top_LI32. They differ with the middle part: one sequence contains PCR_1_Start while the other one contains PCR_2_start. The end region exhibits the same three-part pattern, with SQK-MAP006_Short_Y_Bottom_LI33 at the beginning, followed by either PCR_3_End or PCR_2_End in the middle, and then SQK-NSK007_Y_Bottom at the end. This demonstrates the ability of Porechop_ABI to successfully manage datasets with mixed adapters. To give an idea on the runtime, the execution on this dataset took 70 min on a PC with 16G RAM [Intel(R) Core(TM) i5-3570 CPU] and four threads: 15% of the time is dedicated to ABI preprocessing, and 85% of the time is dedicated to adapter clipping with Porechop. It means that the extra cost of adapter inference is reasonable compared to the whole processing time.

The two last examples of Table 1, cDNA sequencing of *Zea mays* and mitochondrial genome sequencing of *Percina kusha* (the fish bridled darter) are datasets that have each been previously basecalled with Guppy with trimming mode activated. The question is whether there are still adapter traces that could be detected by the program. In both cases, Porechop_ABI was able to extract a signal that corresponds to residual PCR adapters (Fig. 2). For the maize dataset, it appeared that the Porechop_ABI sequence of the start adapter is found in 31% reads in the region [1,150], and of the end adapter in 39% reads in the region [−150,−1]. For the bridled starter, traces of the start adapter are found in 81% reads in the region [1,150]. There is no end adapter detected.

Lastly, we also evaluated the precision of the method by testing the program on negative datasets, which are not supposed to exhibit adapters. The first type of negative datasets is composed of random sequences. The second type of negative datasets is composed of sequencing reads (DNA and cDNA) for which we have removed start and end regions (100 nt). Exhaustive results are presented in Subsection 2.6 of Supplementary Material. In all cases, Porechop_ABI found no motif.

## 5 Discussion

We have developed a new software that meets the initial requirements: to infer the adapter sequences in ONT reads, without any prior knowledge about the adapters. It allows one to determine whether the reads have already been trimmed out, and if not, which adapters have been used. This algorithm is integrated into the open-source software Porechop, which makes it easy to use and allows trimming out in a single pass once the adapters have been identified.

We believe that Porechop_ABI can be useful to help analyzing freshly sequenced data, by verifying that the reads indeed contain the expected adapters or that they have been accurately trimmed out. It also facilitates the usage of data available on public repositories, that often lack metadata.

## Supplementary Material

vbac085_Supplementary_Data
